# Papilledema With Facial and Vestibulocochlear Nerve Involvement in Idiopathic Intracranial Hypertension: An Unusual Case

**DOI:** 10.1155/crop/5103077

**Published:** 2026-09-07

**Authors:** Rosángela López-Mena, Carolina Rojas-Cruz, Fernando Ruiz-Magaña, Eduardo I. Arteaga-Chan

**Affiliations:** ^1^ Department of Ophthalmology, ISSSTE Hospital Regional “General Ignacio Zaragoza”, Mexico City, Mexico; ^2^ National Autonomous University of Mexico (Universidad Nacional Autónoma de México), Mexico City, Mexico, unam.mx

**Keywords:** facial nerve palsy, idiopathic intracranial hypertension, papilledema, sensorineural hearing loss

## Abstract

**Introduction:**

Idiopathic intracranial hypertension (IIH) is defined by elevated intracranial pressure in the absence of an intracranial mass or cerebrospinal fluid abnormality and is commonly associated with papilledema. Although cranial nerve involvement may occur, simultaneous facial and vestibulocochlear nerve dysfunction is rare and may lead to diagnostic confusion.

**Case Presentation:**

A 42‐year‐old obese woman with metabolic comorbidities developed acute right‐sided peripheral facial palsy preceded by facial pain and accompanied by severe headache, pulsatile tinnitus, and blurred vision. A 5‐day course of oral prednisolone for presumed Bell′s palsy yielded no improvement. Ophthalmologic referral on Day 7 revealed bilateral Frisén Grade I papilledema with normal automated perimetry and absence of visual field defects. Neurological examination confirmed House–Brackmann Grade III facial palsy, and audiometry demonstrated bilateral high‐frequency sensorineural hearing loss (right worse than left). After systematic exclusion of cerebellopontine angle lesions, demyelinating disorders, infectious neuritis, and venous sinus thrombosis, brain MRI showed a Grade IV sellar arachnoidocele and a neurovascular contact of the right facial nerve (Chavda Grade I). Lumbar puncture on Day 12 documented an opening pressure of 36 cm H_2_O with normal cerebrospinal fluid, confirming IIH. Acetazolamide was started on Day 13 together with a structured weight‐management program. At the 2‐month follow‐up, audiometry demonstrated complete recovery. By 3 months, papilledema persisted (Frisén Grade I), best corrected visual acuity improved to 20/100 in both eyes, visual fields remained without enlargement of the physiological blind spot, and facial nerve function returned to House–Brackmann Grade I. However, at the 6‐month follow‐up, a new localized inferior scotoma was detected on Humphrey perimetry, representing a progression from previously normal visual fields, whereas spectral‐domain OCT demonstrated bilateral optic atrophy with ganglion cell–inner plexiform layer thinning, indicating irreversible axonal damage despite persistent papilledema.

**Conclusion:**

This case broadens the phenotypic spectrum of IIH, showing that facial and vestibulocochlear neuropathies are reversible with pressure reduction. Yet, progressive visual field loss may ensue despite stable papilledema, revealing the optic disc as an unreliable proxy for neuronal integrity. Only spectral‐domain OCT—by quantifying inner retinal layer loss—can expose silent axonal degeneration and must therefore become the cornerstone of long‐term monitoring, independent of clinical appearance.

**Highlights**

• Idiopathic intracranial hypertension (IIH) can present with concurrent facial and vestibulocochlear nerve dysfunction.
• Initial misdiagnosis as Bell′s palsy may delay crucial treatment.
• Ophthalmologic evaluation with funduscopy is essential in patients with facial palsy and headache.
• Brain MRI and lumbar puncture are diagnostic pillars for confirming IIH.
• Early treatment leads to reversal of cranial neuropathies but may not prevent optic atrophy, underscoring the value of long‐term retinal imaging.

## 1. Case Report

A 42‐year‐old woman with Class III obesity (body mass index, 40.7 kg/m^2^), Type 2 diabetes mellitus, and hypertension presented with acute right‐sided facial pain that evolved over several hours into a peripheral facial palsy with incomplete eyelid closure (Day 0). Simultaneously, she developed severe holocephalic headache, pulsatile tinnitus, and blurred vision. Bell′s palsy was initially suspected, and oral prednisolone (60 mg daily) was initiated on Day 1 for 5 days. Owing to the absence of clinical improvement, she was referred for ophthalmologic assessment on Day 7.

**Figure 1:**
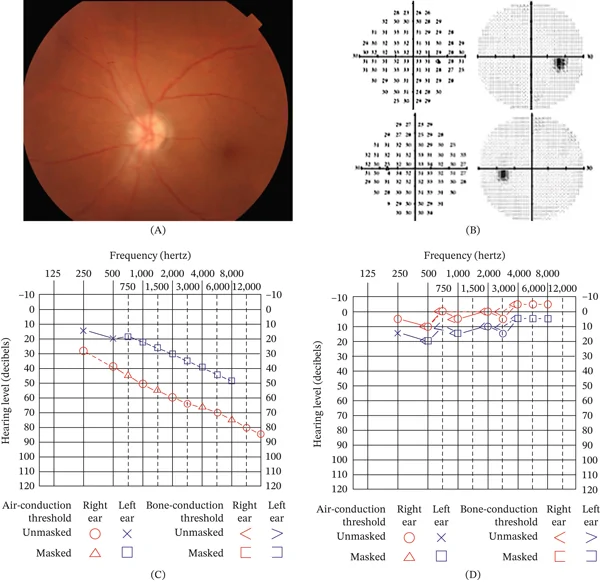
(A) Fundoscopic photograph of the left eye showing optic disc edema consistent with Frisén Grade I papilledema (a similar appearance was present in the right eye). (B) Humphrey visual field (24‐2 SITA‐Fast strategy) demonstrated preserved visual fields without enlargement of the physiological blind spot in either eye. (C, D) Sequential audiometric data documenting a reversible right‐predominant sensorineural hearing loss. The image (C) establishes the initial asymmetry and sensory deficit; the image (D) confirms substantial auditory recovery at the 2‐month follow‐up.

At presentation, glycemic control was optimal (HbA1c 6.2%), and blood pressure was elevated (130/82 mmHg). Funduscopic examination disclosed bilateral papilledema Frisén Grade I (Figure 1), whereas automated perimetry demonstrated preserved visual fields without enlargement of the physiological blind spot in either eye. Best corrected visual acuity was 20/200 in the right eye and 20/400 in the left eye. Neurological examination confirmed a right peripheral facial nerve palsy, corresponding to House–Brackmann Grade III. Audiometric evaluation performed on Day 10 revealed bilateral high‐frequency sensorineural hearing loss, more pronounced on the right side (Figure 1C).

**Figure 2:**
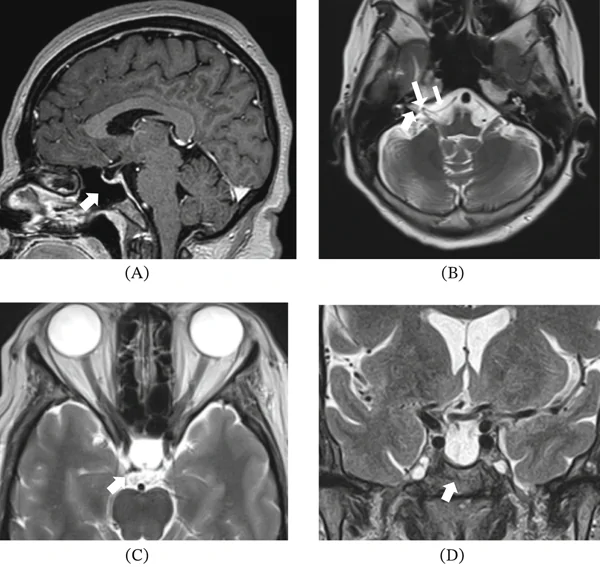
(A) Contrast‐enhanced brain MRI demonstrating a Grade IV sellar arachnoidocele, consistent with an empty sella configuration. (B) T2‐weighted MRI of the cerebellopontine angle showing a right‐sided vascular loop (Chavda Grade I) in close contact with the facial (CN VII) and vestibulocochlear (CN VIII) nerves. The nerves and the adjacent vascular loop are marked with arrows (CN VII, arrowhead; CN VIII, thin arrow; vascular loop, thick arrow). (C) Sagittal T2‐weighted brain MRI demonstrating an empty sella turcica, characterized by CSF filling the sellar cavity with flattening of the pituitary gland. (D) Coronal T2‐weighted brain MRI showing the empty sella with CSF occupying the sella.

Brain magnetic resonance imaging obtained on Day 10 demonstrated no evidence of mass lesions or venous sinus thrombosis but revealed a Grade IV sellar arachnoidocele and a right‐sided neurovascular contact involving the cisternal segment of the facial nerve (Chavda Grade I) (Figure 2).

Lumbar puncture was performed on Day 12 and showed an opening pressure of 36 cm H_2_O with normal cerebrospinal fluid composition, thereby establishing the diagnosis of IIH according to the modified Dandy criteria [1]. All secondary causes of intracranial hypertension were thus excluded, including cerebral venous sinus thrombosis, space‐occupying lesions, infections, and meningeal inflammatory processes, confirming the idiopathic nature of the condition. Acetazolamide (500 mg twice daily) was initiated on Day 13, together with a structured weight‐reduction program.

At the 1‐month follow‐up, headache and pulsatile tinnitus had markedly improved, and facial nerve dysfunction had regressed to House–Brackmann Grade II. At the 2‐month follow‐up, repeat audiometry demonstrated substantial recovery of hearing thresholds, with no clinically significant residual deficit (Figure 1D).

At 3 months after treatment initiation, funduscopic examination demonstrated persistent papilledema (Frisén Grade I), with best corrected visual acuity improving to 20/100 in both eyes. Humphrey automated perimetry continued to show no visual field defects and no enlargement of the physiological blind spot, and facial nerve function had fully normalized (House–Brackmann Grade I), with complete eyelid closure and restoration of facial symmetry.

**Figure 3:**
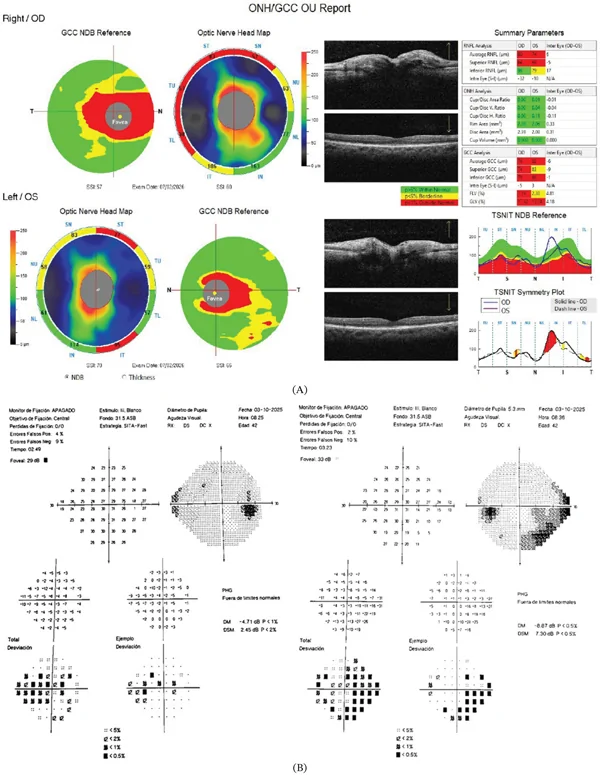
(A) Spectral‐domain OCT (RTVue‐OCT, Optovue Inc.) examination disclosed a bilateral optic atrophy pattern in the setting of resolved papilledema, accompanied by a substantial decrease in the macular ganglion cell complex (GCC) thickness (OD: 68.4 *μ*m; OS: 66.2 *μ*m; normative mean: 95.9 ± 8.9 * μ*m). This structural profile is indicative of a long‐standing, atrophic stage, reflecting permanent and nonrecoverable axonal degeneration. (B) Humphrey visual field (OD, 24‐2 SITA‐Fast). The test discloses a localized, inferiorly predominant scotoma suggestive of glaucomatous optic neuropathy. Reliability parameters (fixation losses, false positives, and false negatives) were all within acceptable clinical ranges.

At the 6‐month follow‐up, Humphrey visual field testing (OD, 24‐2 SITA‐Fast) disclosed the first appearance of a localized inferior scotoma, representing a new functional visual field defect compared with previously normal examinations. Reliability parameters were all within acceptable clinical limits (Figure 3B). Spectral‐domain optical coherence tomography (SD‐OCT) (RTVue‐OCT, Optovue Inc., Fremont, CA, United States) demonstrated bilateral optic atrophy with marked thinning of the macular ganglion cell complex (GCC), with average values of 68.4 *μ*m (OD) and 66.2 *μ*m (OS), consistent with irreversible axonal degeneration despite persistent papilledema (Figure 3A). The patient remained on acetazolamide therapy and continued her structured weight‐management program, achieving a reduction in body mass index from 40.7 to 38.1 kg/m^2^.

## 2. Discussion

After exclusion of alternative etiologies, the clinical presentation was considered most consistent with IIH manifesting as an atypical cranial polyneuropathy involving cranial nerves VII and VIII. Although abducens nerve palsy is a recognized false‐localizing sign of raised intracranial pressure, simultaneous facial and audiovestibular involvement is uncommon [1, 2]. The complete resolution of facial weakness and complete recovery of hearing following intracranial pressure‐lowering therapy strongly support a reversible pressure‐dependent mechanism rather than a primary structural neuropathy.

The concomitant finding of a low‐grade neurovascular conflict involving the facial nerve likely represented a pre‐existing anatomical substrate that remained clinically silent until intracranial pressure increased. Similarly, the presence of a Grade IV sellar arachnoidocele is best interpreted as a radiologic marker of chronic intracranial hypertension rather than a causative lesion [3, 4]. Together, these findings suggest that longstanding intracranial pressure elevation may have preceded the acute presentation, with a subsequent trigger precipitating symptomatic decompensation.

One possible explanation for the abrupt onset of symptoms is a recent increase in body weight. Although weight change could not be objectively documented, rapid weight gain is a recognized precipitating factor for the development and recurrence of IIH and may induce sudden elevations in intracranial pressure [5]. Such a mechanism could account for the unexpectedly acute presentation despite relatively mild papilledema.

A notable feature of this case was the marked reduction in visual acuity despite Frisén Grade I papilledema and initially normal visual fields. In typical IIH, visual acuity is usually preserved until advanced optic nerve involvement develops, as early papilledema predominantly affects the peripapillary retinal nerve fiber layer while relatively sparing central vision [6]. SD‐OCT proved crucial in resolving this apparent discrepancy. Despite the mild funduscopic appearance, OCT demonstrated significant bilateral GCC and ganglion cell–inner plexiform layer thinning, indicating established axonal loss at presentation [7].

Furthermore, longitudinal follow‐up revealed a dissociation between optic disc appearance and neural integrity. While papilledema remained clinically stable and the cranial neuropathies resolved, OCT demonstrated progressive structural optic nerve damage accompanied by the subsequent development of an inferior visual field defect. These findings suggest that retinal ganglion cell degeneration may continue despite apparent stabilization of optic disc edema and initially preserved perimetry [6, 7].

The principal clinical message of this case is that reversible cranial neuropathies may coexist with irreversible optic nerve injury in IIH. Accordingly, normalization of symptoms or stability of papilledema should not be interpreted as evidence of disease quiescence. Longitudinal surveillance with both automated perimetry and OCT is essential to detect ongoing neuroaxonal loss and guide long‐term management [7].

## 3. Conclusion

This case expands the spectrum of cranial nerve manifestations associated with IIH and demonstrates that facial and vestibulocochlear neuropathies may be reversible after intracranial pressure reduction. However, progressive visual field loss from normal baseline to a defined scotoma may occur despite persistent papilledema stability, highlighting the risk of delayed optic nerve damage. Consequently, in chronic IIH, the optic disc appearance is a deceptive proxy for neuronal integrity; only the objective quantification provided by SD‐OCT of the inner retinal layers can unmask silent axonal attrition and must therefore become the indispensable cornerstone of long‐term surveillance, beyond apparent clinical stability.

## Author Contributions

E.I.A‐C. contributed to the conception and design of the case report, data acquisition, analysis, and drafting of the manuscript. F.R‐M. contributed to clinical data interpretation and critical revision of the manuscript. C.R‐C. contributed to the neurological and pathophysiological interpretation of the case and manuscript revision. R.L‐M. contributed to supervision, critical revision for important intellectual content, and final approval of the manuscript.

## Funding

No funding was received for this manuscript.

## Disclosure

All authors read and approved the final manuscript.

## Ethics Statement

This study was conducted in accordance with the principles of the Declaration of Helsinki. Ethical approval was not required for this study, as it is a single case report without experimental intervention, in accordance with the policies of the local Institutional Review Board. Written informed consent was obtained from the patient for the publication of this case report and any accompanying images.

## Conflicts of Interest

The authors declare no conflicts of interest.

## Data Availability

Data sharing is not applicable to this article as no datasets were generated or analyzed during the current study.
